# Hydrophobic Ion Pairing of Small Molecules: How to Minimize Premature Drug Release from SEDDS and Reach the Absorption Membrane in Intact Form

**DOI:** 10.1021/acsbiomaterials.2c01504

**Published:** 2023-02-14

**Authors:** Helen Spleis, Christoph Federer, Victor Claus, Matthias Sandmeier, Andreas Bernkop-Schnürch

**Affiliations:** † Thiomatrix Forschungs- und Beratungs GmbH, Trientlgasse 65, 6020Innsbruck, Austria; ‡ Department of Pharmaceutical Technology, University of Innsbruck, Institute of Pharmacy, Center for Chemistry and Biomedicine, Innrain 80/82, 6020Innsbruck, Austria

**Keywords:** hydrophobic ion pairing, self-emulsifying drug delivery systems, oral drug delivery, small molecule, ethacridine, drug release

## Abstract

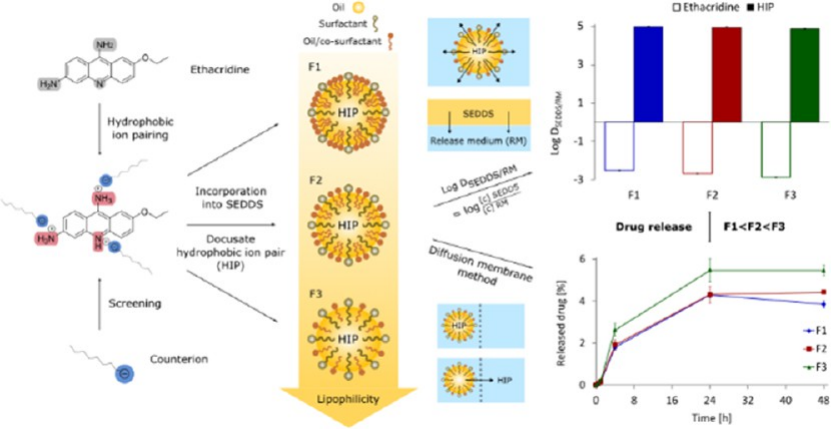

The present work aimed to form hydrophobic ion pairs (HIPs) of a small molecule remaining inside the oily droplets of SEDDS to a high extent. HIPs of ethacridine and various surfactants classified by functional groups of phosphates, sulfates, and sulfonates were formed and precipitation efficiency, log D_*n*-octanol/water_, and solubility in different excipients were investigated. Most lipophilic HIPs were incorporated into SEDDS and evaluated regarding drug release. Docusate HIPs showed the highest increase in lipophilicity with a precipitation efficiency of 100%, a log D_*n*-octanol/water_ of 2.66 and a solubility of 132 mg/mL in *n*-octanol, 123 mg/mL in oleyl alcohol, and 40 mg/mL in medium chain triglycerides. Docusate HIPs were incorporated into three SEDDS of increasing lipophilicity (F1 < F2 < F3) based on medium chain triglycerides, oleyl alcohol, Kolliphor EL, and Tween 80 (F1: 1 + 5 + 2 + 2; F2: 3 + 3 + 2 + 2; F3: 5 + 1 + 4 + 0). Highest achievable payloads ranged from 74.49 mg/mL (F3) to 97.13 mg/mL (F1) and log D_SEDDS/RM_ increased by at least 7.5 units (4.99, F1). Drug release studies via the diffusion membrane method confirmed minor release of docusate HIPs from all SEDDS (<2.7% within 4 h). In conclusion, highly lipophilic HIPs remain inside the oily phase of SEDDS and likely reach the absorption membrane in intact form.

## 1 Introduction

In 2021, the US Food and Drug Administration (FDA) approved a remarkable number of new drugs – 60. Over half of these, more precisely 36, were small molecules. Although the number of approved biologics is constantly increasing since the 1980s, the ratio between biologic and small molecular approvals remains stable at one to two.^1^ The pharmaceutical industry is highly interested in oral delivery systems for small molecules because of their great potential, especially in cancer treatment.^2^ Oral administration is the most preferred route of drug delivery due to its cost-effectiveness, ease of administration and patient compliance. Various small molecules, however, belong to the biopharmaceutical classification system (BCS) class ΙΙΙ characterized by high solubility in gastrointestinal (GI) fluids but low permeability at the absorption membrane. To address the consequently poor oral bioavailability, lipid-based nanocarriers such as self-emulsifying drug delivery systems (SEDDS) were identified as promising vehicles. Due to sufficient stability in GI fluids, enhanced mucus permeation,^3,4^ and protection from extensive presystemic metabolism in the GI tract^5,6^ affecting even small molecules,^7^ SEDDS are able to carry drugs to the absorption membrane in intact form. To increase the lipophilic character of drugs for incorporation into the oily nanocarriers, hydrophobic ion pairing moved into the focus of scientific research. The concept of this technology is based on charged hydrophilic molecules forming ion pairs with oppositely charged surfactants. Due to the lipophilic substructure of the counterion, the resulting uncharged complex becomes water-insoluble and precipitates in aqueous media.^8^

Although only a few *in vivo* studies are available so far, the effectiveness of this technology for oral drug delivery of small molecules has been clearly emphasized. Oral bioavailability of itraconazole, for instance, was 20-fold higher by hydrophobic ion pairing with docusate and incorporation into SEDDS compared to the free base.^9^ Itraconazole, however, belongs to BCS class ΙΙ drugs demonstrating high lipophilicity by nature. *In vivo* proof of concept for BCS class ΙΙΙ small molecules has not been provided so far as the *in vivo* performance of HIPs has not yet reached its full potential. Before even reaching the absorption membrane, HIPs tend to be released from the oily SEDDS droplets and are destroyed in intestinal fluids by competitive endogenous counterions and H-bond donors or acceptors such as electrolytes, bile salts, and fatty acids as well as mucus glycoproteins.^10^ As HIPs can reach the absorption membrane in intact form only when they are shielded from these competitive counterions within the lipophilic phase of SEDDS, it was the aim of this study to form highly lipophilic HIPs remaining inside the oily droplets to a very high extent. To achieve this goal, various types of surfactants were tested for hydrophobic ion pairing and SEDDS were designed without hydrophilic co-solvents such as dimethyl sulfoxide (DMSO), ethanol, benzyl alcohol, propylene glycol, or tetraglycol that were recently identified as main trigger for premature and uncontrolled drug release.^11^ Since hydrophilic organic solvents are immediately released from the oily droplets, HIPs precipitate in SEDDS or follow the co-solvent’s way out of the droplets into the aqueous medium. Beneficial properties of the oily droplets for HIPs such as sufficient solubility and improved stability in the GI tract are no longer provided.

**Figure 1:**
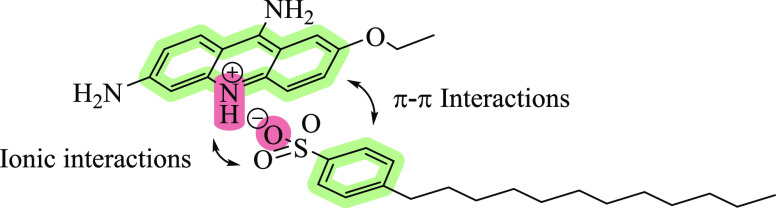
Possible mechanisms of interaction between ethacridine and surfactants using the example of sodium dodecylbenzene sulfonate as counterion.

The small molecule ethacridine was chosen as model drug. Its cationic charge, high level of ionization at neutral pH (p*K*_a_ = 11.04),^12^ and planar surface area providing additional π–π interactions with aromatic structures seem to make it a perfect candidate not just for antimicrobial chemotherapy^13^ but also as small molecule for hydrophobic ion pairing (Figure 1).

Ethacridine offers antimicrobial properties due to intercalation with bacterial DNA and inhibition of protein biosynthesis.^14^ The drug is therefore commonly used as topical wound disinfectant in solutions of 0.1% (Rivanol).^14^ Due to its poor absorption after oral administration (1%),^13^ it is also used for treatment of acute^15^ and chronic^16^ diarrhea (Tannacomp).

## 2 Materials and Methods

### 2.1 Materials

Bis(2-ethylhexyl) phosphate, dibenzyl phosphate, mono-*n*-dodecyl phosphate, *n*-octanol, sodium 1-dodecane sulfonate, sodium *n*-hexadecyl sulfate, sodium *n*-octyl sulfate, sodium 1-pentyl sulfate, and taurocholic acid sodium salt hydrate were purchased from Alfa Aesar (Germany). Sodium 1-octanesulfonic acid sodium salt monohydrate (sodium 1-octane sulfonate) was received from MP Biomedicals (France). 6, 9-Diamino-2-ethoxyacridine-dl-lactate monohydrate (ethacridine lactate salt), dihexadecyl phosphate, 1,2-dioleoyl-sn-glycero-3-phosphate sodium salt (DOPA), 4-(2-hydroxyethyl)piperazine-1-ethanesulfonic acid (HEPES), hexadecyl phosphonic acid, oleyl alcohol, potassium cetyl phosphate, sodium 1-butanesulfonate suitable for ion pair chromatography, sodium docusate (purum), sodium dodecylbenzene sulfonate, sodium 1-heptane sulfonate, sodium hexane sulfonate, and Tween 80 (polyoxyethylene (20) sorbitan monooleate) were obtained from Sigma Aldrich (Austria). Kolliphor EL (Cremophor EL) and medium chain triglycerides (Labrafac lipophile WL 1349) were kind gifts from BASF (Germany) and Gattefossé (France), respectively. SpectraPor Float-A-Lyzer G2 Dialysis Devices (MWCO 3.5–5 kD) were purchased from Lactan (Austria).

### 2.2 Methods

#### 2.2.1 Quantification of Ethacridine by Fluorescence Spectroscopy

Ethacridine was quantified via fluorescence spectroscopy using a microplate reader (TECAN Infinite 200 Pro M Nano^+^, Austria). Calibration curves were established in organic solvents (ethanol, methanol) and aqueous media (25 mM HEPES pH 6.8, water, and a mixture of water and 0.01 M HCl in a ratio of 50/50, v/v). Linearity was confirmed in the range from 0.02 to 1.25 μg/mL for water/0.01 M HCl (50/50, v/v, *R*^2^ = 1.000), from 0.08 to 2.50 μg/mL for 25 mM HEPES pH 6.8 (*R*^2^ = 0.999), and from 0.04 to 2.50 μg/mL for water, ethanol, and methanol (*R*^2^ = 1.000), respectively. Samples of 200 μL were analyzed at an excitation wavelength of 374 nm (ethanol), 372 nm (methanol), or 362 nm (aqueous media) and an emission wavelength of 492 nm (ethanol), 496 nm (methanol), or 514 nm (aqueous media).

#### 2.2.2 Hydrophobic Ion Pairing of Ethacridine

Lipophilic complexes of ethacridine were prepared via hydrophobic ion pairing as previously described by our research group.^17^ Ethacridine was dissolved in 0.01 M HCl at a concentration of 5 mg/mL. Investigated surfactants—classified as phosphates, sulfates, and sulfonates (Table 1)—were dissolved in water at concentrations providing a molar ratio of 1:1. Thereafter, 100 μL of each surfactant solution was added to equal amounts of ethacridine. The immediate appearance of a yellow precipitate indicated HIP formation. After incubation for 30 min at 25 °C and 600 rpm (Vibramax 100, Heidolph Instruments, Germany), HIPs were separated by centrifugation for 15 min at 13,400 rpm (Eppendorf Minispin, Germany). Fluorescence intensity (FI) was measured as described above and precipitation efficiency was calculated using eq 1
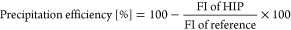
1The resulting HIPs were washed twice with water, dried under vacuum (UniVapo 100 ECH, UniEquip, Germany), and stored at −20 °C until further use.

**Table 1:**
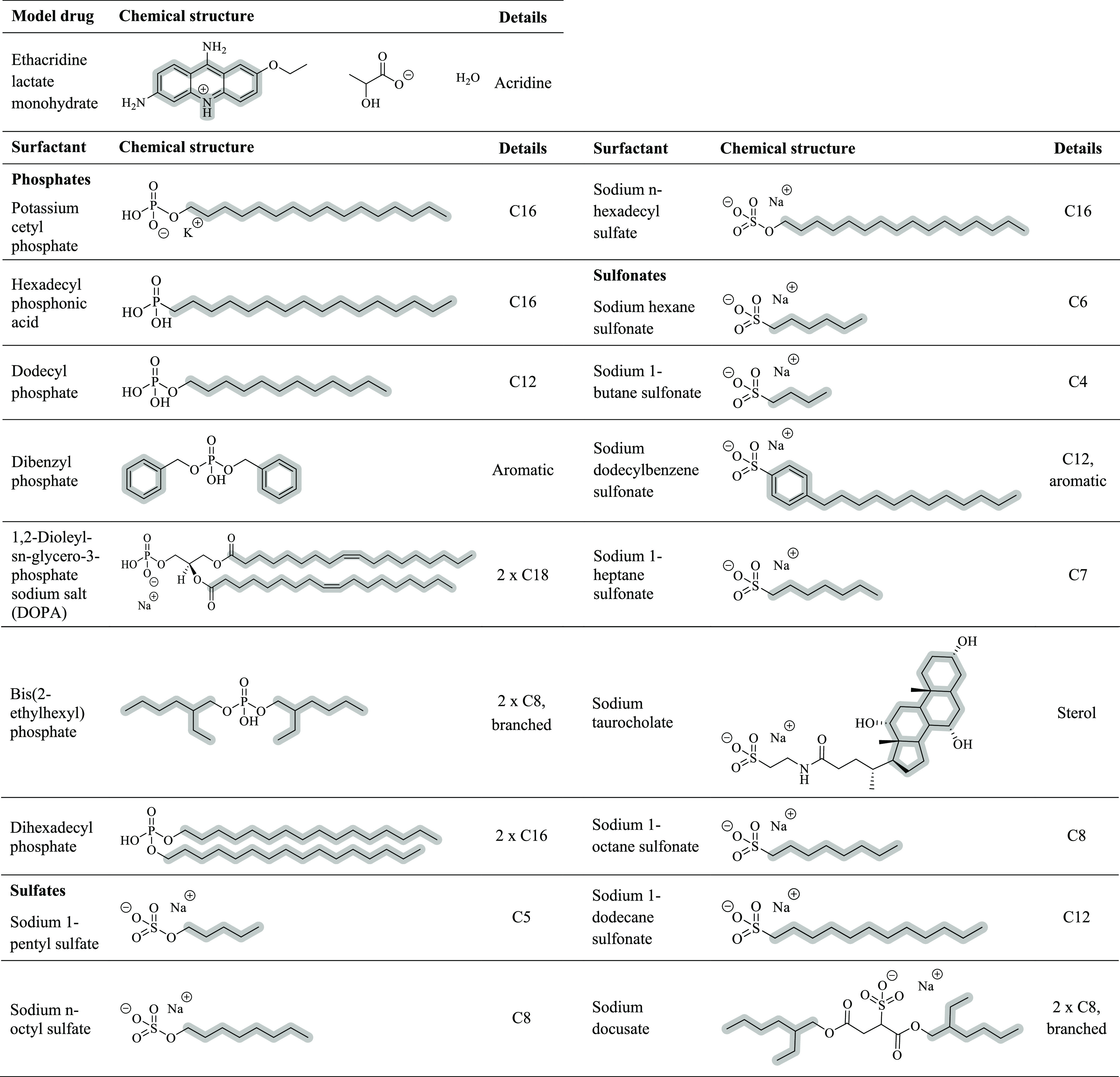
Investigated Surfactants Classified as Phosphates, Sulfates, and Sulfonates for Hydrophobic Ion Pairing of Ethacridine

#### 2.2.3 Determination of log D_*n*-octanol/water_

For determination of the partition coefficient between *n*-octanol and water (log D_*n*-octanol/water_), HIPs as well as reference samples were dispersed in 200 μL of *n*-octanol by ultrasonication for 30 min at 25 °C. Thereafter, 200 μL of water was added and the samples were shaken for 24 h at 25 °C and 600 rpm. After centrifugation for 15 min at 13,400 rpm, both phases were diluted with methanol for fluorescence intensity (FI) measurement as described above. Log D_*n*-octanol/water_ was calculated using eq 2
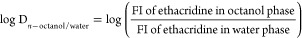
2

#### 2.2.4 Solubility Studies

The increase in solubility of ethacridine after hydrophobic ion pairing was investigated in medium chain triglycerides, *n*-octanol, and oleyl alcohol. HIPs were prepared as described above with minor modifications. In brief, 400 μL of aqueous surfactant solution was added to 400 μL of ethacridine solution in 1.5 mL Eppendorf vessels of conical shape. Thereafter, 40 μL of medium chain triglycerides, *n*-octanol, or oleyl alcohol were added with a positive-displacement pipette to obtain an oversaturated mixture. In case of sodium dodecylbenzene sulfonate and sodium docusate, 750 μL of both solutions were combined to increase the amount of HIP and 25 μL of medium chain triglycerides, *n*-octanol, or oleyl alcohol were added. For comparison, an excess of non-ion paired ethacridine serving as reference was dispersed in medium chain triglycerides, *n*-octanol, and oleyl alcohol. After ultrasonication for 30 min at 25 °C and shaking for 24 h at 25 °C and 600 rpm, the samples were centrifuged for 15 min at 13,400 rpm and the supernatant was diluted with ethanol for quantification of ethacridine as described above.

#### 2.2.5 Preparation and Characterization of SEDDS

Based on the results of preliminary solubility studies, three novel SEDDS formulations of increasing lipophilicity were developed (F1 < F2 < F3, Table 2). Oily components were medium chain triglycerides and oleyl alcohol, which additionally acted as co-surfactant. Kolliphor EL (PEG-35 castor oil) and Tween 80 (polyoxyethylene (20) sorbitan monooleate) were used as surfactants. Excipients were mixed as listed in Table 2 with a vortex mixer and homogenized by ultrasonication.

**Table 2:** Excipients Used for SEDDS Development (* Oil, ** Oil/Co-surfactant, *** Surfactant) and Composition of SEDDS Formulations [%, v/v] (HLB: Hydrophilic–Lipophilic Balance)

		SEDDS formulation		
Excipient	HLB	F1	F2	F3
Medium chain triglycerides * (Labrafac lipophile WL 1349)	1	10	30	50
Oleyl alcohol **	14	50	30	10
Kolliphor EL *** (PEG-35 castor oil)	12–14	20	20	40
Tween 80 *** (Polyoxyethylene (20) sorbitan monooleate)	16.7–17	20	20	

Stability of SEDDS preconcentrates was evaluated visually regarding phase separation after centrifugation for 10 min at 13,400 rpm. For emulsification, 10 μL of SEDDS preconcentrate was diluted with 990 μL of 25 mM HEPES pH 6.8 (37 °C). Droplet size, PDI, and zeta potential of resulting emulsions were analyzed directly after emulsification and after 4 h of incubation at 37 °C and 300 rpm (Digital heating shaking drybath, Thermo Fisher, USA) with a NanoBrook 90Plus PALS (Brookhaven Instruments, USA).

Based on the results of preliminary solubility studies, docusate HIPs were incorporated into SEDDS preconcentrates. Complexes were prepared as described above and dispersed in 25 μL of corresponding SEDDS preconcentrate by ultrasonication for 30 min at 25 °C and shaking for 24 h at 25 °C and 600 rpm. After centrifugation for 15 min at 13,400 rpm, 10 μL of the supernatant was added to 990 μL of 25 mM HEPES pH 6.8 (37 °C). Droplet size, PDI, and zeta potential of loaded SEDDS were evaluated after 0 and 4 h of incubation at 37 °C and 300 rpm.

#### 2.2.6 Evaluation of Drug Release

Drug release behavior of ethacridine-docusate HIPs was characterized by the distribution coefficient (log D_SEDDS/RM_) between the lipophilic phase (SEDDS preconcentrate) and the aqueous phase (release medium, RM) in a separate manner. Log D_SEDDS/RM_ is basically the measurement of maximum solubility in both phases and thus a theoretical evaluation of drug release.^18^ For comparison purposes, drug release was additionally evaluated by a diffusion membrane model where oily droplets and release medium are separated by a semipermeable membrane.

##### 2.2.6.1 Determination of Maximum Solubility and log D_SEDDS/RM_

Log D_SEDDS/RM_ of non-ion paired ethacridine and docusate HIPs was determined by measuring the maximum solubility in SEDDS preconcentrate and release medium separately.^19^ Docusate HIPs were prepared as described above and dispersed in 25 μL of corresponding SEDDS preconcentrate (F1, F2, F3) or 25 mM HEPES pH 6.8 used as release medium. An excess of non-ion paired ethacridine used as reference was dispersed in both systems for comparison. After ultrasonication for 30 min at 25 °C and shaking for 24 h at 600 rpm, the samples were centrifuged for 15 min at 13,400 rpm and the supernatant was diluted with ethanol in case of SEDDS preconcentrates or water in case of release medium. Maximum solubility corresponding to the highest achievable payload was determined by fluorescence spectroscopy as described above and log D_SEDDS/RM_ was calculated using eq 3
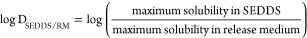
3Furthermore, the concentration of docusate HIPs (*C*_SEDDS_ [%]) remaining inside the oily droplets upon emulsification was calculated using eq 4
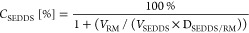
4where *V*_RM_ and *V*_SEDDS_ represent the volume of the release medium and SEDDS preconcentrate, respectively, and D_SEDDS/RM_ the distribution of HIPs in SEDDS preconcentrate and release medium.^18^

##### 2.2.6.2 Diffusion Membrane Method

Drug release behavior of docusate HIPs from SEDDS formulation F1, F2, and F3 was further investigated by a diffusion membrane model. In brief, the oily nanoemulsions were separated from the release medium by a semipermeable membrane. Loaded SEDDS were prepared as described above. Non-ion paired ethacridine dissolved in the aqueous phase at concentrations representing a 100% drug release from SEDDS served as reference. Accordingly, ethacridine was dissolved in 990 μL of 25 mM HEPES pH 6.8 (37 °C) at a concentration of 0.97 mg/mL in case of F1, 0.90 mg/mL for F2, and 0.74 mg/mL for F3, and 10 μL of blank SEDDS preconcentrate was added. Thereafter, 1 mL of sample or control emulsion was transferred into dialysis tubes (SpectraPor Float-A-Lyzer G2 Dialysis Device MWCO 3.5–5 kD) and dialyzed against 9 mL of 25 mM HEPES pH 6.8 in 50 mL falcon tubes while shaking at 300 rpm and 37 °C. After 0.5, 1, 4, 24, and 48 h of incubation, aliquots of 400 μL were removed from the release medium and replaced by fresh buffer (37 °C). For quantification of released ethacridine as described above, samples were diluted with 25 mM HEPES pH 6.8 if required. The amount of ethacridine in the release medium after 48 h served as 100% value.

#### 2.2.7 Statistical Data Analysis

All data are shown as mean of at least three experiments ± standard deviation (SD). Statistical data analyses were performed using Student’s *t*-test with *p* < 0.05 for significant (*), *p* < 0.01 for very significant (**), and *p* < 0.001 for highly significant (***).

## 3 Results and Discussion

### 3.1 Hydrophobic Ion Pairing of Ethacridine

**Figure 2:**
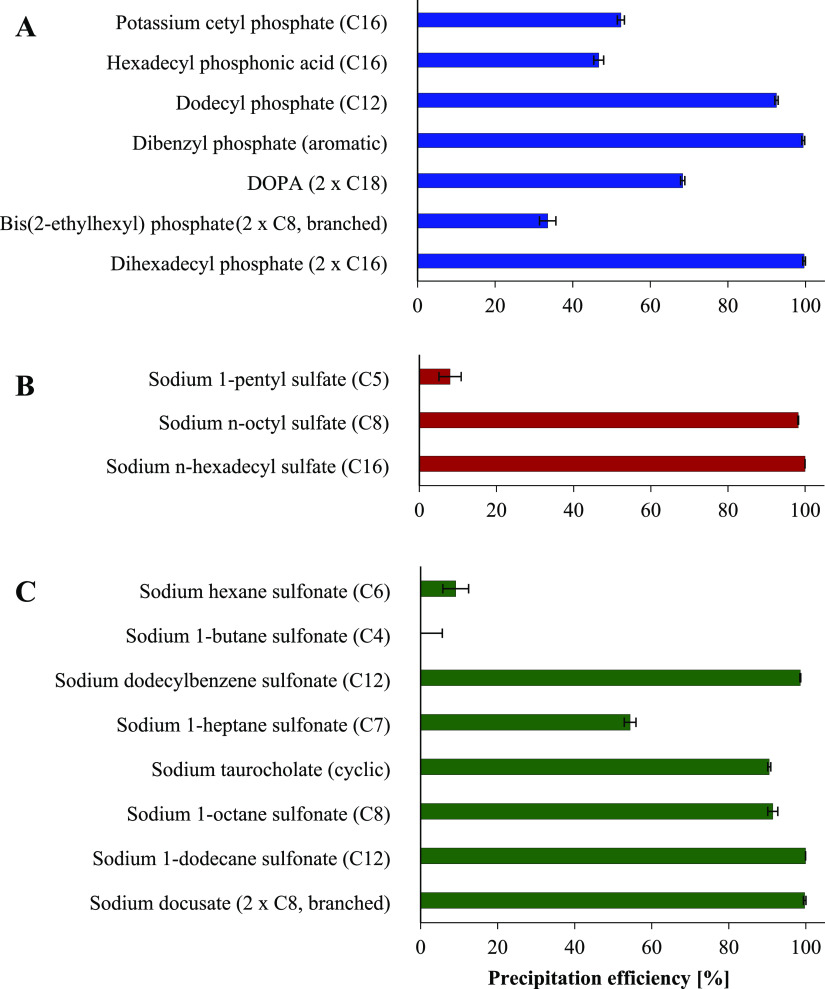
Precipitation efficiency [%] of ethacridine ion-paired with different surfactants classified by functional groups of phosphates (A), sulfates (B), and sulfonates (C). Indicated values are means (*n* ≥ 3) ± SD.

To increase the lipophilicity of ethacridine via hydrophobic ion pairing, various counterions classified by functional groups of phosphates, sulfates, and sulfonates, were screened. Their potential to raise the lipophilic character of the drug was evaluated based on their precipitation efficiency (Figure 2). Among tested phosphates, the highest precipitation efficiencies ≥99% were determined for dibenzyl phosphate (aromatic) most likely based on π–π interactions between the two planar systems, and for dihexadecyl phosphate providing two lipophilic carbon chains (2 × C16). In comparison, potassium cetyl phosphate and hexadecyl phosphonic acid with just one carbon chain of equal length (C16) demonstrated significantly lower complex formation efficiencies <53%. Thus, surfactants with two carbon chains precipitated ethacridine to a higher extent compared to counterions exhibiting only one lipophilic tail of equal length. Besides the number of carbon chains, their length was identified as key parameter. Sodium *n*-hexadecyl sulfate (C16) and sodium *n*-octyl sulfate (C8), for instance, showed precipitation efficiencies >98%, whereas sodium 1-pentyl sulfate (C5) demonstrated minor potential evidenced by a precipitation efficiency of 8 ± 3%. Similar observations were made for investigated sulfonates. Surfactants providing carbon chain lengths ≥C8 such as sodium 1-octane sulfonate (C8), sodium dodecylbenzene sulfonate (C12, aromatic), and sodium 1-dodecane sulfonate (C12) precipitated >90% of ethacridine, whereas significantly lower precipitation efficiencies of 54 ± 2% and 9 ± 3% were demonstrated with counterions having chain lengths <C8 such as sodium 1-heptane sulfonate (C7) and sodium hexane sulfonate (C6), respectively.

### 3.2 Determination of log D_*n*-octanol/water_

**Figure 3:**
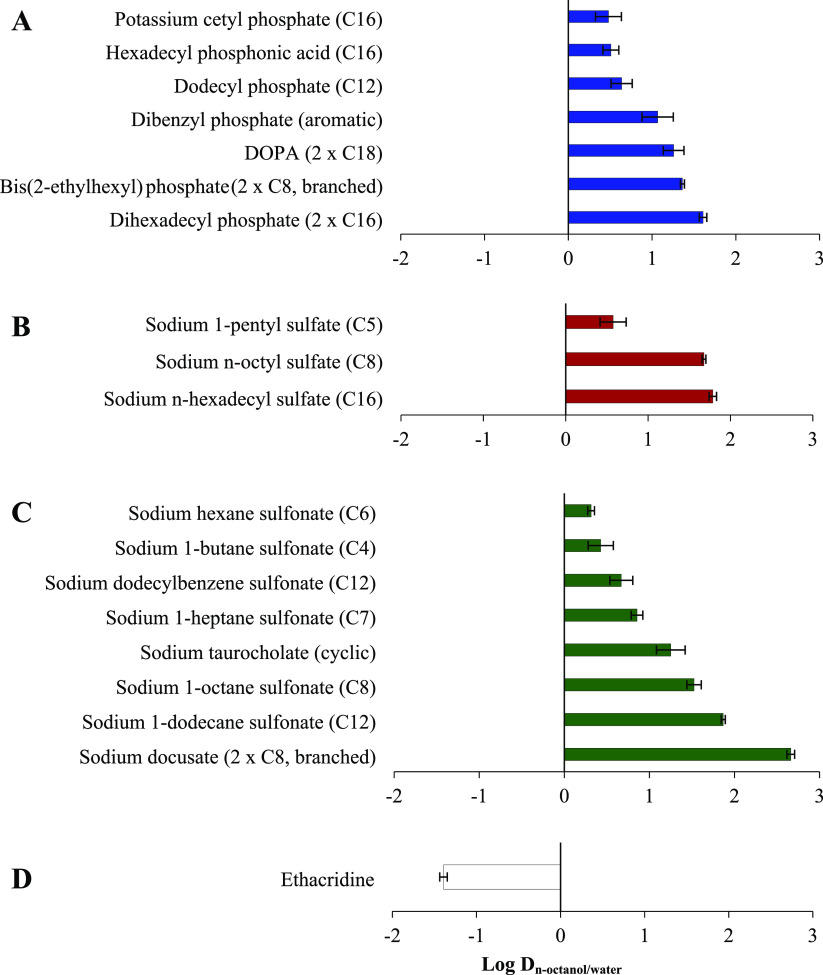
Log D_*n*-octanol/water_ of ethacridine after hydrophobic ion pairing with different surfactants classified by functional groups of phosphates (A), sulfates (B), and sulfonates (C) compared to non-ion paired ethacridine used as reference (D). Indicated values are means (*n* ≥ 3) ± SD.

The distribution of HIPs between *n*-octanol and water was determined to evaluate the increase in lipophilicity of ethacridine via hydrophobic ion pairing in comparison to non-ion paired drug. As shown in Figure 3, all surfactants provided an increase in log D_*n*-octanol/water_ compared to native ethacridine with a log D_*n*-octanol/water_ of −1.39 ± 0.02. In particular, counterions with two carbon chains such as dihexadecyl phosphate (2 × C16), bis(2-ethylhexyl) phosphate (2 × C8), and DOPA (2 × C18) raised the lipophilicity of ethacridine to a high extent (log D_*n*-octanol/water_ ≥1.26). The superior role of surfactants providing two lipophilic alkyl tails in comparison with one tail of equal length was further evidenced by the 3-fold higher log D_*n*-octanol/water_ of dihexadecyl phosphate (2 × C16, 1.61 ± 0.12) compared to potassium cetyl phosphate (C16, 0.48 ± 0.14). Moreover, surfactants providing branched carbon chains were advantageous for hydrophobic ion pairing of ethacridine. HIPs with bis(2-ethylhexyl) phosphate (2 × C8, branched), for instance, showed sufficient increase in lipophilicity (log D_*n*-octanol/water_ = 1.36 ± 0.19) despite low precipitation efficiency (34 ± 2%). Considering the structural similarity to the gold standard counterion sodium docusate,^20^ the effectiveness of branched surfactants was further highlighted. Another key parameter of counterions to raise the lipophilic character of ethacridine was their alkyl chain length. Sulfate-based HIPs, for instance, demonstrated higher log D_*n*-octanol/water_ values ≥1.68 for C16- and C8-chain bearing surfactants such as sodium *n*-hexadecyl sulfate and sodium *n*-octyl sulfate compared to sodium 1-pentyl sulfate with C5-carbon chain length and a log D_*n*-octanol/water_ of 0.58 ± 0.15. However, HIPs with sodium dodecylbenzene sulfonate providing sufficient alkyl chain length (C12, aromatic) demonstrated inappropriate correlation between precipitation efficiency (99%) and log D_*n*-octanol/water_ (0.67 ± 0.04). This discrepancy can be explained by the high water solubility of the complex. Log D_*n*-octanol/water_ values > 1 among sulfonate-based HIPs were observed for sodium taurocholate (cyclic, log D_*n*-octanol/water_ = 1.25 ± 0.13) and surfactants having chain lengths ≥C8 such as sodium 1-octane sulfonate (C8, log D_*n*-octanol/water_ = 1.52 ± 0.07) and sodium 1-dodecane sulfonate (C12, log D_*n*-octanol/water_ = 1.87 ± 0.17). The highest log D_*n*-octanol/water_ of 2.66 ± 0.08 among all tested counterions was achieved with sodium docusate that perfectly meets the abovementioned requirements with two branched alkyl tails (2 × C8). This result is in good accordance with identified key features of sulfosuccinate-based counterions for hydrophobic ion pairing of peptides and proteins by Wibel et al. who demonstrated the superior role of branched alkyl tails compared to linear ones.^20^ These findings underline again the immense potential of sodium docusate as lead counterion as well as the superior role of sulfosuccinate-based surfactants for hydrophobic ion pairing in general.

### 3.3 Solubility Studies

**Figure 4:**
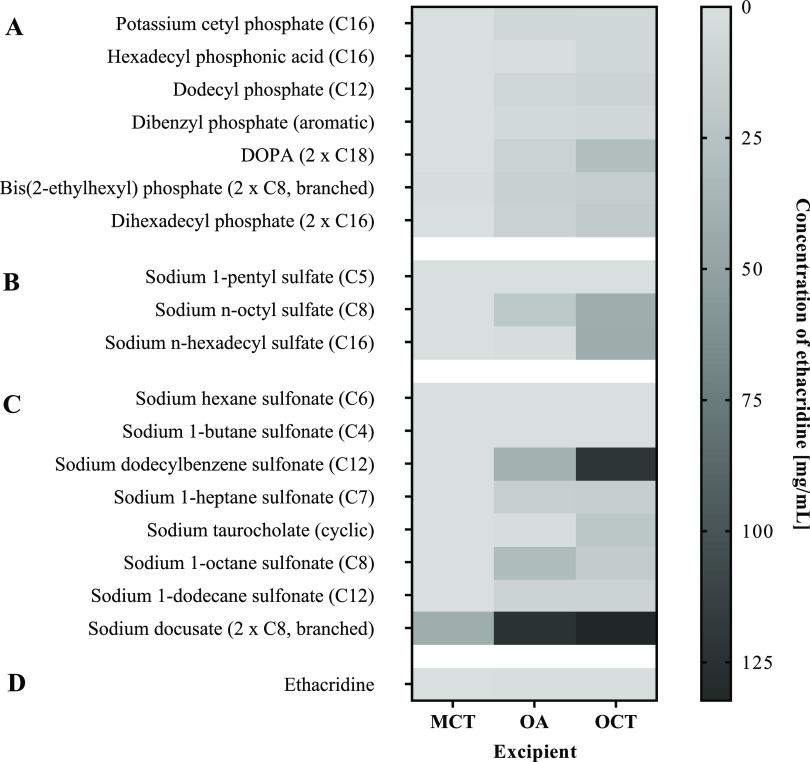
Solubility in medium chain triglycerides (MCT, Labrafac lipophile WL 1349), oleyl alcohol (OA), and *n*-octanol (OCT) of ethacridine after hydrophobic ion pairing with different surfactants classified by functional groups of phosphates (A), sulfates (B), and sulfonates (C) compared to non-ion paired ethacridine used as reference (D). Indicated values are means (*n* ≥ 3) ± SD.

The increase in solubility of ethacridine after hydrophobic ion pairing was investigated in *n*-octanol and in commonly used excipients for SEDDS development such as medium chain triglycerides and oleyl alcohol (Figure 4). In particular, ethacridine complexes based on sulfonate surfactants demonstrated high solubility. Dodecylbenzene sulfonate HIPs, for instance, were highly soluble in *n*-octanol (120 ± 1 mg/mL) and oleyl alcohol (37 ± 5 mg/mL) but insoluble in medium chain triglycerides (<0.1 mg/mL). The highest solubility among all investigated HIPs was observed for docusate HIPs with concentrations of 132 ± 4 mg/mL in *n*-octanol, 123 ± 3 mg/mL in oleyl alcohol, and 40 ± 2 mg/mL in medium chain triglycerides. Non-ion paired ethacridine, in contrary, was minor soluble in *n*-octanol (1.04 ± 0.07 mg/mL), oleyl alcohol (1.25 ± 0.18 mg/mL), and medium chain triglycerides (0.00 ± 0.00 mg/mL). In summary, docusate HIPs were 127-fold (*n*-octanol), 98-fold (oleyl alcohol), and 8 957-fold (medium chain triglycerides) higher soluble in investigated excipients than the reference. Based on these results, the solubility of ethacridine was effectively increased via hydrophobic ion pairing. Docusate HIPs were regarded as most promising and safe for oral administration as the surfactant is listed in the inactive ingredient database of the FDA. Docusate HIPs were therefore further evaluated in SEDDS formulations.

### 3.4 Preparation and Characterization of SEDDS

Based on the results of preliminary solubility studies, docusate HIPs were incorporated into three SEDDS formulations of increasing lipophilicity (F1 < F2 < F3, Table 2) to investigate the influence on drug release. The oily phase consisting of medium chain triglycerides was continuously increased from 10% in F1 to 30% in F2 and 50% in F3. Accordingly, F3 showed higher droplet size (122.1 ± 2.2 nm) than F1 (76.5 ± 4.1 nm) and F2 (68.3 ± 1.1 nm). After incorporation of docusate HIPs, droplet sizes increased in case of all SEDDS formulations confirming successful incorporation of HIPs. The highest increase in droplet size was observed for F1 which might be explained by the highest amount of oleyl alcohol providing highest solubility of docusate HIPs. Blank as well as loaded SEDDS formulations were stable over 4 h as no pronounced changes in droplet size were observed. Zeta potential was constantly negative over 4 h of incubation ranging from −2.7 ± 0.5 mV to −8.8 ± 0.5 mV. In general, SEDDS exhibiting small droplet size (<200 nm) and negative zeta potential provide advantages such as higher mucus permeability than SEDDS with larger droplet size and positive zeta potential. According to Griesser et al., a decrease in droplet size results in an increase in permeation rate.^3^ The smaller the SEDDS, the higher their mucus permeating properties were. Droplet sizes of 25, 50, and 100 nm demonstrated higher mucus permeating properties (≥6.17%) compared to higher droplet sizes of 200 and 500 nm (<6%). Furthermore, the mesh size of mucus between 20 and 200 nm emphasized the importance of a droplet size <200 nm for sufficient permeation as particles >200 nm demonstrated minor permeation properties.^21^ An overview of droplet size, PDI, and zeta potential of blank and loaded SEDDS formulations is provided in Table 3.

**Table 3:** Droplet Size, Polydispersity Index (PDI), and Zeta Potential of Blank and Loaded (*Italics*) SEDDS after Emulsification to 1% (v/v) with 25 mM HEPES pH 6.8. Indicated Values Are Means (*n* ≥ 3) ± SD

F	Droplet size [nm] after 0 / 4 h	PDI after 0 / 4 h	Zeta potential [mV] after 0 / 4 h
1	76.5 ± 4.1 / 73.8 ± 2.9	0.23 ± 0.01 / 0.24 ± 0.01	–5.2 ± 0.8 / –5.4 ± 0.9
	*122.1 ± 5.6* / *115.1 ± 6.4*	*0.33 ± 0.01* / *0.33 ± 0.02*	*–7.4 ± 0.8* / *–5.2 ± 1.1*
2	68.3 ± 1.1 / 66.8 ± 0.9	0.15 ± 0.01 / 0.14 ± 0.03	–4.3 ± 0.8 / –5.5 ± 1.2
	*81.8 ± 10.6* / *78.1 ± 8.9*	*0.17 ± 0.04* / *0.16 ± 0.03*	*–8.3 ± 1.6* / *–5.8 ± 1.8*
3	122.1 ± 2.2 / 119.6 ± 2.4	0.14 ± 0.01 / 0.13 ± 0.02	–2.7 ± 0.5 / –4.0 ± 0.6
	*129.7 ± 5.6* / *131.5 ± 1.8*	*0.26 ± 0.02* / *0.31 ± 0.01*	*–8.8 ± 0.5* / *–5.5 ± 1.8*

### 3.5 Evaluation of Drug Release

#### 3.5.1 Determination of Maximum Solubility and log D_SEDDS/RM_

**Figure 5:**
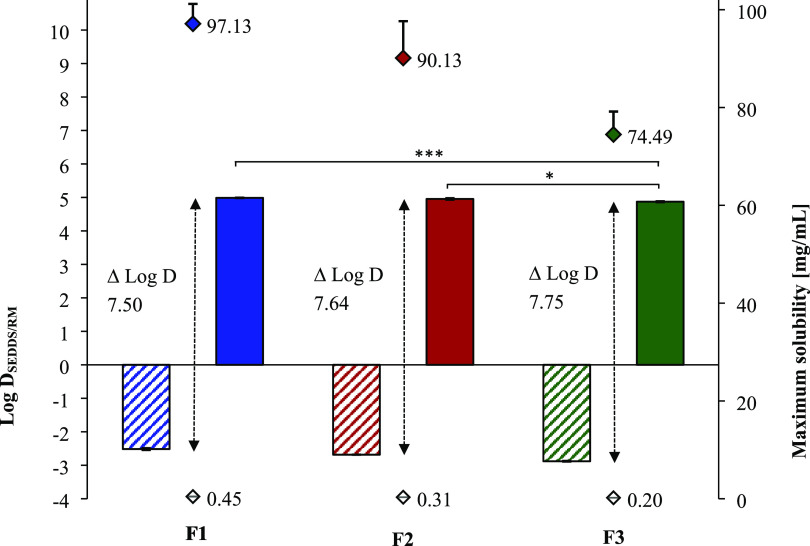
Maximum solubility of non-ion paired ethacridine (◊) used as reference and ethacridine-docusate HIPs (⧫) in SEDDS formulations F1, F2, and F3. Log D values between SEDDS formulation F1, F2, F3 and 25 mM HEPES pH 6.8 used as release medium (RM) of ethacridine (hatched bars) as well as ethacridine-docusate HIPs (solid bars). Difference in Log D_SEDDS/RM_ between ethacridine and ethacridine-docusate HIPs shown as Δ Log D. Indicated values are means (*n* ≥ 3) ± SD (*p < 0.05, ***p < 0.001).

To guarantee sufficient incorporation of HIPs into SEDDS formulations, the maximum solubility of docusate HIPs in F1, F2, and F3 was determined. Results indicated a clear correlation between maximum solubility and the amount of oleyl alcohol in corresponding SEDDS formulation (F1 > F2 > F3) as the fatty alcohol provided the highest solubility of docusate HIPs among tested solvents. In fact, F1 demonstrated maximum solubility of docusate HIPs (97.13 ± 4.07 mg/mL), followed by F2 (90.13 ± 7.53 mg/mL) and F3 (74.49 ± 4.68 mg/mL, Figure 5). So far, Griesser et al. demonstrated comparable payloads (>100 mg/mL) by hydrophobic ion pairing of leuprolide, insulin, and desmopressin with sodium docusate.^17^ These results, however, are primarily attributable to the presence of hydrophilic co-solvents such as Transcutol HP, tetraglycol, and propylene glycol in SEDDS up to 30%. Hydrophilic co-solvents are well known to improve the solubility of HIPs in SEDDS. After emulsification in intestinal fluids, however, they are immediately released from the oily droplets causing drug precipitation in SEDDS on the one hand and triggering drug release into the aqueous phase on the other hand.^11^ Hydrophobic ion pairing has been widely used for incorporation of BCS class ΙΙΙ proteins and peptides into SEDDS for oral drug delivery, whereas small molecules were mainly unreported so far. Patel et al. incorporated >10% of lumefantrine into SEDDS after hydrophobic ion pairing with oleic acid.^22^ Morgen et al. increased the solubility of atazanavir in SEDDS from 0.75 mg/mL to 2.80 mg/mL and 4.30 mg/mL by hydrophobic ion pairing with 2-naphthalene sulfonate and sodium docusate, respectively.^23^ Both small molecules, however, are BCS class ΙΙ drugs of high lipophilicity by nature. Native ethacridine, in contrast, is highly water-soluble belonging to BCS class ΙΙΙ. The 215- (F1), 294- (F2), and 382-fold (F3) higher solubility of ethacridine-docusate HIPs than non-ion paired ethacridine (solubility <0.5 mg/mL) in SEDDS without hydrophilic co-solvents underlines their superior role over previously designed HIPs with small molecules.

Compared to solid lipid-based formulations where drug release can be tuned by multiple mechanisms,^24,25^ sufficient drug solubility in liquid SEDDS is one of the key parameters to keep and stabilize HIPs inside the oily droplets and to prevent premature drug release upon emulsification in physiological media. Furthermore, stability of HIPs is provided as competitive counterions such as electrolytes, bile salts, or anionic mucus substructures are too hydrophilic to enter the lipophilic phase.^5^ Despite these benefits, however, the potential of SEDDS is limited by uncontrolled and premature drug release based on a simple diffusion process from the lipophilic liquid phase into the aqueous liquid phase.^18^ Drug molecules in the oily droplets diffuse to the droplet’s surface, overcome the interfacial barrier, and finally reach the aqueous medium. Due to the submicron size of the droplets, HIPs rapidly move out of the oily droplets until equilibrium between SEDDS and release medium is reached—usually within seconds.^18^ Besides the solubility of HIPs in the lipophilic phase, their affinity to the aqueous medium is of great relevance. So far, drug release studies were challenging as the oily droplets need to be separated from the aqueous phase for quantification of the drug in the release medium. A more straightforward strategy to evaluate the release behavior of drugs from SEDDS is the determination of their distribution coefficient (log D_SEDDS/RM_) between lipophilic phase (SEDDS preconcentrate) and aqueous phase (release medium, RM) as described in detail previously.^18^ Following this approach, drug release is just the measurement of solubility in SEDDS preconcentrate and release medium in a separate manner. In general, a log D_SEDDS/RM_ <3 results in immediate and high drug release.^18^ Retaining drugs inside the oily droplets, therefore, requires a log D_SEDDS/RM_ >3. Chamieh et al., for instance, reported that 100% of leuprolide-docusate HIPs with a log D_SEDDS/RM_ of 3 remained inside the oily droplets, whereas only 30% remained inside in case of desmopressin-docusate HIPs having a log D_SEDDS/RM_ of 0.5.^26^ Similar observations were made by Bonengel et al. where octreotide-decanoate HIPs with a log D_SEDDS/RM_ of 1.7 showed faster release than octreotide-docusate HIPs exhibiting a log D_SEDDS/RM_ of 2.7.^27^ In our study, ethacridine-docusate HIPs demonstrated log D_SEDDS/RM_ values >4.8 (Figure 5) indicating that nearly 100% (*C*_SEDDS_ >99.8%) of the drug will remain inside the oily droplets upon emulsification with intestinal fluids. The highest log D_SEDDS/RM_ of 4.99 ± 0.02 was reached in F1, followed by F2 and F3 with log D_SEDDS/RM_ values of 4.95 ± 0.04 and 4.87 ± 0.03, respectively. Determined log D_SEDDS/RM_ values were in good accordance with maximum solubilities of docusate HIPs in corresponding SEDDS formulations as highest payload yielded highest log D_SEDDS/RM_. Non-ion paired ethacridine showed log D_SEDDS/RM_ values in the range of −2.52 ± 0.04 (F1) to −2.88 ± 0.02 (F3). The highest increase in log D_SEDDS/RM_ of 7.75 units compared to the reference (shown as Δ log D in Figure 5) was demonstrated for F3, the most lipophilic SEDDS formulation containing 50% of medium chain triglycerides.

#### 3.5.2 Diffusion Membrane Method

**Figure 6:**
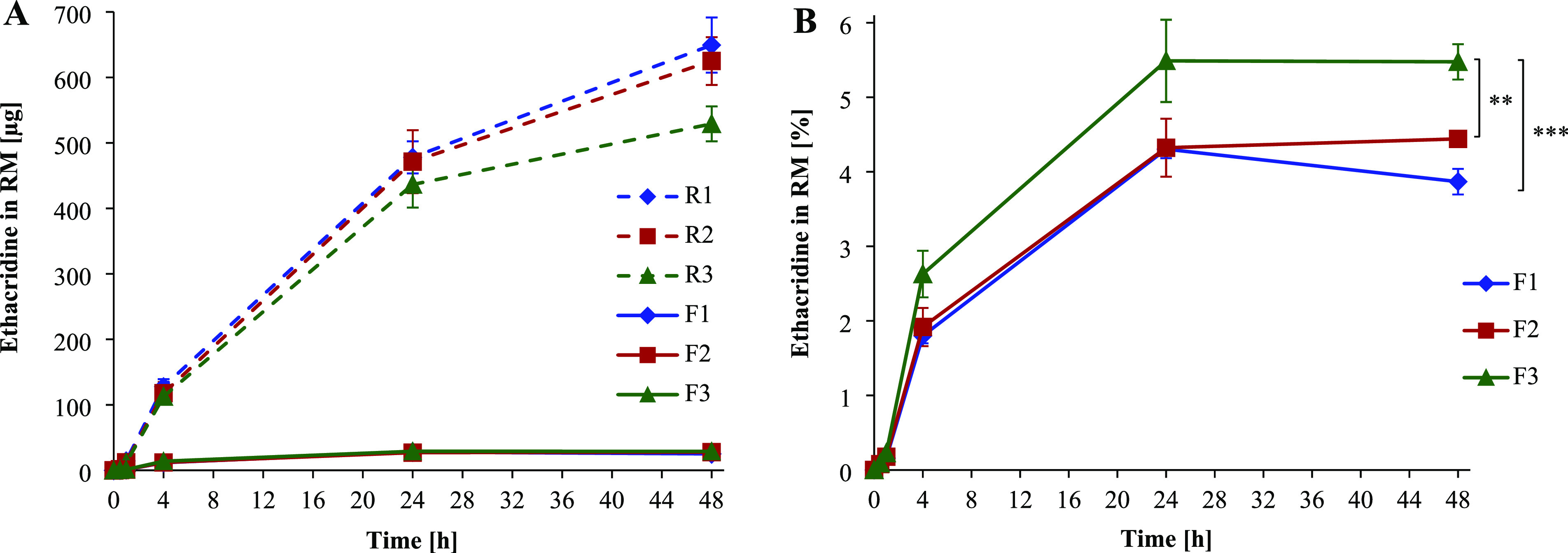
(A) Ethacridine [μg] released from SEDDS formulation F1, F2, and F3 compared to reference R1, R2, and R3 (dissolution of ethacridine in release medium at concentrations providing a 100% drug release (0.97 mg/mL for F1, 0.90 mg/mL for F2, and 0.74 mg/mL for F3) and subsequent addition of blank SEDDS preconcentrate for emulsification). (B) Ethacridine [%] released from SEDDS formulation F1, F2, and F3. Released ethacridine of reference emulsions after 48 h served as 100% value. 1 mL of emulsion was dialyzed against 9 mL of release medium (RM, 25 mM HEPES pH 6.8) at 37 °C under shaking at 300 rpm. Indicated values are means (*n* = 3) ± SD (**p < 0.01, ***p < 0.001).

Since log D_SEDDS/RM_ values are calculated from maximum solubilities of the drug in SEDDS preconcentrate and aqueous phase, release kinetics of ethacridine from SEDDS formulation F1, F2, and F3 were further investigated by a diffusion membrane model. Oily droplets and release medium were thereby separated by a dialysis membrane that is permeable to free drug but impermeable to oily droplets. So far, control experiments were primarily performed with aqueous drug solutions omitting SEDDS formulations and their impact on drug release.^28,29^ In this study, ethacridine was pre-dissolved in release medium at concentrations representing the worst-case scenario of a 100% drug release (0.97 mg/mL for F1, 0.90 mg/mL for F2, and 0.74 mg/mL for F3). Thereafter, the corresponding blank SEDDS preconcentrate was added for emulsification. The amount of ethacridine quantified in the release medium after 48 h was used as 100% value. As shown in Figure 6A, 25–29 μg of ethacridine was released from SEDDS after 48 h, whereas in case of control emulsions 529–649 μg of the drug was quantified in the release medium. This corresponds to a drug release of 3.9 ± 0.2% for F1, 4.4 ± 0.1% for F2, and 5.5 ± 0.2% for F3 after 48 h (Figure 6B). Since pharmaceutical dosage forms pass through the GI tract for approximately 4 h before absorption, premature drug release during this transit time needs to be avoided. As shown in Figure 6B, all SEDDS demonstrated minor release of ethacridine after 4 h (<2.7%). The lowest drug release was observed for F1 (1.8 ± 0.1%), followed by F2 (1.9 ± 0.3%) and F3 (2.6 ± 0.3%) representing a clear correlation with determined log D_SEDDS/RM_ values (F1 > F2 > F3).

Based on these results, HIPs likely reach the absorption membrane in intact form. To predict oral drug absorption *in vivo*, permeability studies using Caco-2 cell monolayers^30^ or rat intestinal mucosa mounted in Ussing chambers^31^ have emerged as standard *in vitro* tools and could therefore be used as potential absorption screening models in further studies.

## 4 Conclusions

The present study aimed to raise the lipophilic character of the small molecule ethacridine via hydrophobic ion pairing. For this purpose, various surfactants were investigated and classified by functional groups of phosphates, sulfates, and sulfonates. Their chain length and the number of lipophilic alkyl tails were identified as key features since counterions with carbon chains ≥C8 or two lipophilic tails increased the lipophilicity of ethacridine to a higher extent than surfactants with chain lengths <C8 or only one lipophilic tail of equal length. Furthermore, counterions providing branched tails were superior over linear ones. In general, sulfonates yielded most lipophilic HIPs in comparison to phosphate- and sulfate-based HIPs. In particular sodium docusate effectively increased the lipophilicity of ethacridine due to its two branched C8-carbon chains that perfectly match previously mentioned requirements. Docusate HIPs were therefore incorporated into three novel SEDDS formulations of increasing lipophilicity (F1 < F2 < F3). Log D_SEDDS/RM_ values increased by at least 7.5 units (>4.8) in comparison to non-ion paired drug, indicating that HIPs remain inside the oily droplets upon emulsification with intestinal fluids to a very high extent. Drug release studies via a diffusion membrane model confirmed these results. All SEDDS formulations provided sufficient stability of HIPs by minor drug release (F1 < F2 < F3). According to these results, it is feasible to design highly lipophilic HIPs of small molecules that remain in the oily droplets of SEDDS and therefore reach the absorption membrane most likely in intact form.

## References

[ref1] Kinch M. S.; Kraft Z.; Schwartz T. 2021 in Review: FDA Approvals of New Medicines. Drug Discovery Today 2022, 27, 2057–2064. 10.1016/j.drudis.2022.04.010. 35439613

[ref2] Zhong L.; Li Y.; Xiong L.; Wang W.; Wu M.; Yuan T.; Yang W.; Tian C.; Miao Z.; Wang T.; Yang S. Small Molecules in Targeted Cancer Therapy: Advances, Challenges, and Future Perspectives. Signal Transduct. Target. Ther. 2021, 6, 201 10.1038/s41392-021-00572-w. 34054126 PMC8165101

[ref3] Griesser J.; Hetényi G.; Kadas H.; Demarne F.; Jannin V.; Bernkop-Schnürch A. Self-Emulsifying Peptide Drug Delivery Systems: How to Make Them Highly Mucus Permeating. Int. J. Pharm. 2018, 538, 159–166. 10.1016/j.ijpharm.2018.01.018. 29339247

[ref4] Abdulkarim M.; Sharma P. K.; Gumbleton M. Self-Emulsifying Drug Delivery System: Mucus Permeation and Innovative Quantification Technologies. Adv. Drug Delivery Rev. 2019, 142, 62–74. 10.1016/j.addr.2019.04.001. 30974131

[ref5] Hetényi G.; Griesser J.; Moser M.; Demarne F.; Jannin V.; Bernkop-Schnürch A. Comparison of the Protective Effect of Self-Emulsifying Peptide Drug Delivery Systems towards Intestinal Proteases and Glutathione. Int. J. Pharm. 2017, 523, 357–365. 10.1016/j.ijpharm.2017.03.027. 28347848

[ref6] Liu J.; Hirschberg C.; Fanø M.; Mu H.; Müllertz A. Evaluation of Self-Emulsifying Drug Delivery Systems for Oral Insulin Delivery Using an in Vitro Model Simulating the Intestinal Proteolysis. Eur. J. Pharm. Sci. 2020, 147, 105272 10.1016/j.ejps.2020.105272. 32084584

[ref7] Pereira de Sousa I.; Bernkop-Schnürch A. Pre-Systemic Metabolism of Orally Administered Drugs and Strategies to Overcome It. J. Controlled Release 2014, 192, 301–309. 10.1016/j.jconrel.2014.08.004. 25128718

[ref8] Ristroph K. D.; Prud’homme R. K. Hydrophobic Ion Pairing: Encapsulating Small Molecules, Peptides, and Proteins into Nanocarriers. Nanoscale Adv. 2019, 1, 4207–4237. 10.1039/C9NA00308H. 33442667 PMC7771517

[ref9] Sahbaz Y.; Williams H. D.; Nguyen T.-H.; Saunders J.; Ford L.; Charman S. A.; Scammells P. J.; Porter C. J. H. Transformation of Poorly Water-Soluble Drugs into Lipophilic Ionic Liquids Enhances Oral Drug Exposure from Lipid Based Formulations. Mol. Pharm. 2015, 12, 1980–1991. 10.1021/mp500790t. 25905624

[ref10] Nazir I.; Asim M. H.; Dizdarević A.; Bernkop-Schnürch A. Self-Emulsifying Drug Delivery Systems: Impact of Stability of Hydrophobic Ion Pairs on Drug Release. Int. J. Pharm. 2019, 561, 197–205. 10.1016/j.ijpharm.2019.03.001. 30836151

[ref11] Jörgensen A. M.; Friedl J. D.; Wibel R.; Chamieh J.; Cottet H.; Bernkop-Schnürch A. Cosolvents in Self-Emulsifying Drug Delivery Systems (SEDDS): Do They Really Solve Our Solubility Problems?. Mol. Pharm. 2020, 17, 3236–3245. 10.1021/acs.molpharmaceut.0c00343. 32658482 PMC7482394

[ref12] Albert A.; Goldacre R. The Ionisation of Acridine Bases. J. Chem. Soc. 1945, 706, 706–713. 10.1039/jr9460000706 20282440

[ref13] Wainwright M. Acridine--a Neglected Antibacterial Chromophore. J. Antimicrob. Chemother. 2001, 47, 1–13. 10.1093/jac/47.1.1. 11152426

[ref14] Junka A.; Bartoszewicz M.; Smutnicka D.; Secewicz A.; Szymczyk P. Efficacy of Antiseptics Containing Povidone-Iodine, Octenidine Dihydrochloride and Ethacridine Lactate against Biofilm Formed by Pseudomonas Aeruginosa and Staphylococcus Aureus Measured with the Novel Biofilm-Oriented Antiseptics Test. Int. Wound J. 2014, 11, 730–734. 10.1111/iwj.12057. 23445335 PMC7950748

[ref15] Ericsson C. D. Nonantimicrobial Agents in the Prevention and Treatment of Traveler’ Diarrhea. Clin. Infect. Dis. 2005, 41, S557–S563. 10.1086/432952. 16267719

[ref16] Plein K.; Burkard G.; Hotz J. Treatment of Chronic Diarrhea in Crohns Disease. A Pilot Study of the Clinical Effect of Tannin Albuminate and Ethacridine Lactate. Fortschr. Med. 1993, 111, 114–118. 8462917

[ref17] Griesser J.; Hetényi G.; Moser M.; Demarne F.; Jannin V.; Bernkop-Schnürch A. Hydrophobic Ion Pairing: Key to Highly Payloaded Self-Emulsifying Peptide Drug Delivery Systems. Int. J. Pharm. 2017, 520, 267–274. 10.1016/j.ijpharm.2017.02.019. 28188875

[ref18] Bernkop-Schnürch A.; Jalil A. Do Drug Release Studies from SEDDS Make Any Sense?. J. Controlled Release 2018, 271, 55–59. 10.1016/j.jconrel.2017.12.027. 29287908

[ref19] Shahzadi I.; Dizdarević A.; Efiana N. A.; Matuszczak B.; Bernkop-Schnürch A. Trypsin Decorated Self-Emulsifying Drug Delivery Systems (SEDDS): Key to Enhanced Mucus Permeation. J. Colloid Interface Sci. 2018, 531, 253–260. 10.1016/j.jcis.2018.07.057. 30036849

[ref20] Wibel R.; Knoll P.; Le-Vinh B.; Kali G.; Bernkop-Schnürch A. Synthesis and Evaluation of Sulfosuccinate-Based Surfactants as Counterions for Hydrophobic Ion Pairing. Acta Biomater. 2022, 144, 54–66. 10.1016/j.actbio.2022.03.013. 35292415

[ref21] Leonaviciute G.; Zupančič O.; Prüfert F.; Rohrer J.; Bernkop-Schnürch A. Impact of Lipases on the Protective Effect of SEDDS for Incorporated Peptide Drugs towards Intestinal Peptidases. Int. J. Pharm. 2016, 508, 102–108. 10.1016/j.ijpharm.2016.04.044. 27143595

[ref22] Patel K.; Sarma V.; Vavia P. Design and Evaluation of Lumefantrine – Oleic Acid Self Nanoemulsifying Ionic Complex for Enhanced Dissolution. DARU J. Pharm. Sci. 2013, 21, 27 10.1186/2008-2231-21-27. PMC3635930 23531442

[ref23] Morgen M.; Saxena A.; Chen X.-Q.; Miller W.; Nkansah R.; Goodwin A.; Cape J.; Haskell R.; Su C.; Gudmundsson O.; Hageman M.; Kumar A.; Chowan G. S.; Rao A.; Holenarsipur V. K. Lipophilic Salts of Poorly Soluble Compounds to Enable High-Dose Lipidic SEDDS Formulations in Drug Discovery. Eur. J. Pharm. Biopharm. 2017, 117, 212–223. 10.1016/j.ejpb.2017.04.021. 28438550

[ref24] Joyce P.; Dening T. J.; Meola T. R.; Schultz H. B.; Holm R.; Thomas N.; Prestidge C. A. Solidification to Improve the Biopharmaceutical Performance of SEDDS: Opportunities and Challenges. Adv. Drug Delivery Rev. 2019, 142, 102–117. 10.1016/j.addr.2018.11.006. 30529138

[ref25] Müller R. H.; Mäder K.; Gohla S. Solid Lipid Nanoparticles (SLN) for Controlled Drug Delivery - a Review of the State of the Art. Eur. J. Pharm. Biopharm. 2000, 50, 161–177. 10.1016/S0939-6411(00)00087-4. 10840199

[ref26] Chamieh J.; Domènech Tarrat A.; Doudou C.; Jannin V.; Demarne F.; Cottet H. Peptide Release from SEDDS Containing Hydrophobic Ion Pair Therapeutic Peptides Measured by Taylor Dispersion Analysis. Int. J. Pharm. 2019, 559, 228–234. 10.1016/j.ijpharm.2019.01.039. 30703502

[ref27] Bonengel S.; Jelkmann M.; Abdulkarim M.; Gumbleton M.; Reinstadler V.; Oberacher H.; Prüfert F.; Bernkop-Schnürch A. Impact of Different Hydrophobic Ion Pairs of Octreotide on Its Oral Bioavailability in Pigs. J. Controlled Release 2018, 273, 21–29. 10.1016/j.jconrel.2018.01.012. 29355620

[ref28] Karamanidou T.; Karidi K.; Bourganis V.; Kontonikola K.; Kammona O.; Kiparissides C. Effective Incorporation of Insulin in Mucus Permeating Self-Nanoemulsifying Drug Delivery Systems. Eur. J. Pharm. Biopharm. 2015, 97, 223–229. 10.1016/j.ejpb.2015.04.013. 25933940

[ref29] Zaichik S.; Steinbring C.; Menzel C.; Knabl L.; Orth-Höller D.; Ellemunter H.; Niedermayr K.; Bernkop-Schnürch A. Development of Self-Emulsifying Drug Delivery Systems (SEDDS) for Ciprofloxacin with Improved Mucus Permeating Properties. Int. J. Pharm. 2018, 547, 282–290. 10.1016/j.ijpharm.2018.06.005. 29883790

[ref30] van Breemen R. B.; Li Y. Caco-2 Cell Permeability Assays to Measure Drug Absorption. Expert Opin. Drug Metab. Toxicol. 2005, 1, 175–185. 10.1517/17425255.1.2.175. 16922635

[ref31] Föger F.; Kopf A.; Loretz B.; Albrecht K.; Bernkop-Schnürch A. Correlation of in Vitro and in Vivo Models for the Oral Absorption of Peptide Drugs. Amino Acids 2008, 35, 233–241. 10.1007/s00726-007-0581-5. 17726639

